# Chemically Induced pH Perturbations for Analyzing Biological Barriers Using Ion-Sensitive Field-Effect Transistors

**DOI:** 10.3390/s21217277

**Published:** 2021-11-01

**Authors:** Tatsuro Goda

**Affiliations:** Department of Biomedical Engineering, Faculty of Science and Engineering, Toyo University, 2100 Kujirai, Kawagoe, Saitama 350-8585, Japan; goda@toyo.jp; Tel.: +81-49-239-1746

**Keywords:** potentiometry, label-free, cell membranes, tight junctions, cytotoxicity

## Abstract

Potentiometric pH measurements have long been used for the bioanalysis of biofluids, tissues, and cells. A glass pH electrode and ion-sensitive field-effect transistor (ISFET) can measure the time course of pH changes in a microenvironment as a result of physiological and biological activities. However, the signal interpretation of passive pH sensing is difficult because many biological activities influence the spatiotemporal distribution of pH in the microenvironment. Moreover, time course measurement suffers from stability because of gradual drifts in signaling. To address these issues, an active method of pH sensing was developed for the analysis of the cell barrier in vitro. The microenvironmental pH is temporarily perturbed by introducing a low concentration of weak acid (NH_4_^+^) or base (CH_3_COO^−^) to cells cultured on the gate insulator of ISFET using a superfusion system. Considering the pH perturbation originates from the semi-permeability of lipid bilayer plasma membranes, induced proton dynamics are used for analyzing the biomembrane barriers against ions and hydrated species following interaction with exogenous reagents. The unique feature of the method is the sensitivity to the formation of transmembrane pores as small as a proton (H^+^), enabling the analysis of cell–nanomaterial interactions at the molecular level. The new modality of cell analysis using ISFET is expected to be applied to nanomedicine, drug screening, and tissue engineering.

## 1. Introduction

Proton (hydronium ion) is involved in many essential biological reactions, such as the equilibrium of carbonate ions, glycolysis, and enzymatic reactions. Systemic pH level has long been recognized as a typical sign of the homeostatic condition. In fact, altered pH is closely related to pathological conditions, such as tumor growth, bacterial infection, and dental caries [1,2,3]. As a result, various sensing techniques have been developed for biological pH measurements, including implantable sensors, positron emission tomography (PET) imaging, magnetic resonance imaging (MRI), and electron paramagnetic resonance (EPR) imaging [4,5,6,7,8]. These techniques have shown the ability to provide semi-quantitative information during in vivo studies, although the accuracy has been difficult to confirm [9]. In recent years, pH sensing has been applied for bioassays and bioanalytical systems [10,11].

The pH monitoring in culture media or extracellular space is useful for noninvasively determining the conditions of cells. The microenvironmental pH gradually decreases with cellular metabolites, including carbon dioxide and lactate; therefore, the acidification rate in the extracellular medium represents the degree of cellular activity [12]. For accurate quantitative sensing, a potentiometric ion-sensitive field-effect transistor (ISFET) array with a perfusion system was developed to measure the acidification rate of extracellular pH for determining the respiration and glycolysis of tumor cells adhered to the gate insulator [13,14,15]. ISFETs have been used for pH sensing and biosensing for decades [16,17,18,19]. The Nernst response at the solution/insulator interface is the main mechanism for potentiometric pH-sensitivity. ISFETs attract attention as a compact, label-free, real-time, high-throughput, and non-cytotoxic biosensing platform because they are manufactured by the complementary MOS (CMOS) process. Therefore, ISFET-based approaches are straightforward for miniaturized multi-parallel biosensing on a small chip [20]. Moreover, the incorporation of a selective layer on the gate insulator surface can extend the applicability to other biosensing targets [21,22,23]. A surface coating with an ion-selective membrane can provide the signal selectivity to physiological ions [24,25,26]. The gate potential of ISFET also responds to microenvironmental changes at the solution/gate interface caused by cell detachment or cell morphology changes on the gate insulator. As a result, an acute cellular response to cytotoxic reagents was estimated using ISFET [14,27].

Recently, a new pH-sensing method was developed to evaluate biological barriers, such as biomembranes and intercellular junctions. Analyzing the biological barriers with high sensitivity, specificity, and spatiotemporal resolutions is essential in the advancement of bioengineering and nanomedicine. The review paper describes a novel potentiometric pH sensing method with the aid of external chemical stimuli and their applications regarding the label-free sensing of unique cellular processes, such as biomembrane injury and epithelial barrier breakdown.

## 2. Analysis of Cell Barriers for Nanomedicine

Spatiotemporal control of the delivery of therapeutic agents to a specific site is going to be realized by a nanobiotechnology-based drug delivery system (DDS), i.e., nanomedicine [28]. DDS applications include cancer immunotherapy, gene therapy, and nucleic acid-based vaccination, such as messenger RNA (mRNA) vaccines against coronavirus disease 2019 (COVID-19) [29]. Anticancer drugs or therapeutic agents are administered in complexation with nanocarriers for enhancing the safety, stability, and targetability in biological conditions [30]. In other words, nanocarriers are intentionally designed to carry payload for maximizing the therapeutic efficacy while minimizing side effects. To this end, an important challenge is overcoming biological barriers without compromising body defense systems [31].

A eukaryotic cell protects itself by its self-assembled lipid bilayer plasma membrane with a thickness of 6–10 nm. A biomembrane is semipermeable; water and small neutral molecules are permeable by passive diffusion; however, charged species and electrolytes are impermeable because of the hydrophobic core of the bilayer [32]. Macromolecules are usually taken up by cells in a series of energy-dependent mechanisms, called endocytosis [33]. Representative nanocarriers are liposomes, polymeric micelles, and inorganic nanoparticles from natural and synthetic origins, whose typical size ranges from tens to a few hundred nanometers. These nanocarriers are usually endocytosed by cells and entrapped in endosomal compartments after internalization. Therefore, therapeutic agents, which are designed to function in the cell organelle, have to escape through the endosomal biomembranes to enhance the drug efficacy. Many efforts have been made for facilitating endosomal escape using the microenvironmental changes associated with lysosomal digestion known as cell autophagy. Another opportunity is that nanocarriers may bypass the endocytic pathways to reach the cytosol by directly permeating through plasma membranes. Some cationic and amphiphilic nanocarriers permeabilize biomembranes by making tiny transmembrane pores or altering the lipid bilayer polarity during internalization [34]. The permeation mechanisms by different nanocarriers are not completely understood because of the lack of sensing techniques for analyzing the interaction between biomembranes and nanocarriers with high spatiotemporal resolutions.

Biological barriers are also found in epithelial/endothelial tissues. Epithelial cells can exert a rigorous barrier function by forming a multi-protein network in the cell gaps called tight junctions (TJs) [35,36,37]. Proteins, such as claudin, occludin, and zonula occludens (ZO), are the main constituents of TJs. These junctions are underpinned by the cytoskeleton via transmembrane proteins in the lateral biomembranes. TJs seal peripherals in the top of apical domain so that solutes and water molecules cannot freely permeate the basolateral side through the paracellular pathways. Epithelial barriers are essential for vertebrates to protect the interior against viral and microbial challenges from the external world. TJs form a selective channel for small ions and water by altering the subtype of the constituent proteins, allowing homeostatic maintenance in epithelial tissues, and nutrition uptake in digestive tracts. Therefore, TJs could be a potential drug discovery target for curing malabsorption, dermatitis, and inflammatory diseases [38]. In DDS, epithelial tissues, such as skin and the mucous membrane, are a convenient drug administration route. Epithelial barriers need to be partially and temporarily breached to increase drug permeability, while also creating safety issues. *Clostridium perfringens* enterotoxin (CPE)-derived TJ-binder was used for promoting mucosal absorption and for cancer targeting in nanomedicine [39]. Neural tissues have a clear boundary to blood circulation, namely the blood-brain barrier (BBB). The interface is composed of endothelial cells with cell–cell junctions including TJs. Overcoming the BBB is essential for nanomedicines to cure brain pathologies and neural diseases. Receptor-mediated transcytosis or induced TJ-loosening is a major route for nanocarriers to translocate across the BBB [40]. Moreover, most malignant tumors originate from epithelial cells. During cancer progression, epithelial cells undergo phenotypic changes termed epithelial-mesenchymal transition (EMT) [41]. EMT includes the loss of epithelial cell–cell junctions including TJs, enabling the cells to invade into neighboring tissues and initiate metastasis. Revealing the regulatory mechanisms of the epithelial barrier transitions in tumor microenvironments will guide the development of new cancer therapies.

There are several ways for evaluating biological barriers in vitro. Measuring the leakage of a biomembrane-impermeable indicator from cell cytosol after challenges by nanocarriers is a common method for investigating biomembrane injuries. Indicators include small fluorescence dyes (e.g., calcein) and proteins (e.g., lactate dehydrogenase (LDH), hemoglobin) [42,43]. Leakage assays are frequently used as cytotoxicity assays because large-scale biomembrane lysis leads to acute necrosis. Although the assays are simple, are applicable to various cell types, and have high throughput, they are difficult to use to characterize the permeation mechanisms of nanocarriers. Specifically, traditional indicators cannot pass through smaller transmembrane pores because of the molecular sieve effect. This is crucial because some nanocarriers are suspected to enter cytoplasm by creating pores at molecular levels. Moreover, some indicators can permeate a biomembrane whose hydrophobic/hydrophilic balance is altered by interacting with nanocarriers [44]. These phenomena cause false-positive and false-negative signals for analyzing nanocarrier-biomembrane interactions. The patch clamp technique can electrically determine cell barrier properties by monitoring ionic currents across the biomembrane at the suction area of a single cell using a micropipette [45]. This method is sensitive because the current represents the diffusion of physiological electrolytes of low molecular weight through damaged biomembranes. However, the method can only analyze a single cell at a time and it requires skilled technicians and custom equipment. Therefore, a novel method that can determine biological barriers with high sensitivity, selectivity, and throughput is needed. 

A new analytical technique that measures the leakage of proton through a damaged biomembrane was proposed [46]. The pH changes in the cell microenvironment were caused by nanocarrier-induced biomembrane damage. Details on this method are described in the latter sections of this paper.

## 3. Weak Acid/Base-Induced pH Perturbation in a Cell Microenvironment

The pH-responsive ISFET-based sensors have been successfully used for noninvasively determining the growth, metabolism, and physiological conditions of cells and bacteria [47,48,49,50]. The acidification rate of the extracellular microenvironment is an important indicator of live cell state [15,51]. The pH changes with the flux of acidic/basic substances via membrane transporters on the oocyte were estimated by the potentiometric responses of an ISFET-microfluidics system [52].

Although passive potentiometric sensing of extracellular pH is simple, it has some drawbacks. First, ISFET-based potentiometric sensing inevitably has a gradual signal drift over time, which needs to be compensated for by a reference signal for accurate measurement [21]. Second, the pH changes are caused by many factors including respiration, metabolism, transporters, adhesion/detachment, and morphological changes. As a result, it is laborious to identify the cause of the signal. To address these issues, an active method of pH sensing, in which the dynamic response in pH is measured following external physical or biochemical stimuli to the cells, occasionally using a fluidic system, has been reported [53,54]. Compared with passive pH sensing, active pH sensing can acquire a specific signal of interest without interference by the homeostatic activities of cells. In addition, the active method avoids signal drift because the pH perturbation upon external stimuli occurs in a short period of time, typically < 1 min. Adaptation of the active sensing into cellular pH measurements introduces a method for better understanding live cell functions. The effect of an amiloride inhibitor for sodium/hydrogen exchangers (NHE) expressed on the surface of live mammalian cells cultured on an ISFET was successfully evaluated by recording dynamic pH changes induced by intervals of ammonia loading and unloading [55].

**Figure 1:**
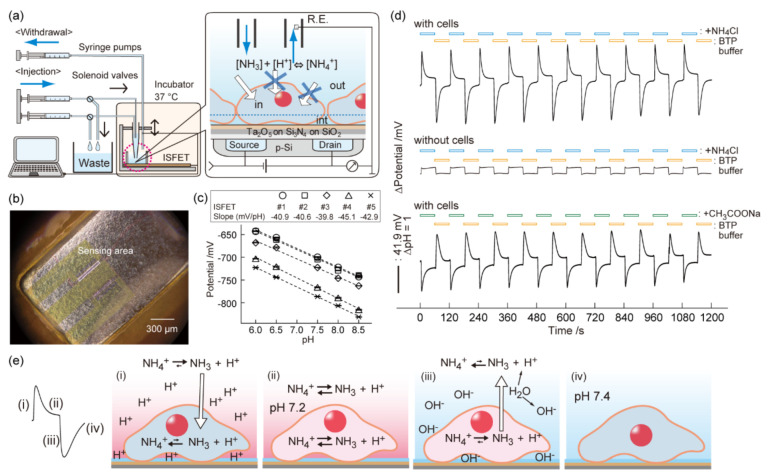
Cell/ISFET system. (**a**) HepG2 cells were cultured on the poly-L-lysine-coated gate insulator of the pH-sensing transistor at subconfluent levels. An automated fluidic system comprising syringe pumps and solenoid valves achieved instant exchange of the solutions surrounding the cells. The inset shows the mass transfer of NH_4_^+^, NH_3_, and H^+^ between the bulk phase, cells/ISFET interspace, and intracellular compartment, respectively, and the ammonia equilibrium reaction in each phase. The semi-permeability of the cell membranes prevented the passive diffusion of charged species into the cells. (**b**) Phase contrast image of HepG2 cells on the gate insulator. (**c**) The Nernst pH response of the ISFET with HepG2 monolayers (*n* = 5). (**d**) Time course of the ISFET potential during periodic flushes (1 min each) of isotonic buffers containing 10 mM (NH_4_Cl), (CH_3_COONa), or 20 mM (sucrose). A pH overshoot occurred when the buffer solution surrounding the cells was exchanged stepwise. The direction was opposite for CH_3_COONa. No pH overshoots occurred in the absence of cells on the gate insulator. (**e**) Schematic illustrations explaining the mechanism of local pH changes during the periodic flushes of NH_4_Cl in the extracellular space. Reproduced with modification from [46] with permission by Elsevier.

A weak acid or base, such as a buffer solution containing ammonium chloride or sodium acetate, are effective pH oscillators in the cell microenvironment (Figure 1) [46,55]. In physiology, a weak acid is a traditional manipulator of cytosolic pH via the proton sponge effect [56]. An extracellular pH gradient was generated by a temporary non-equilibrium state of acid-base reaction as a result of semi-permeable mass transport between the cell interior and exterior. For example, upon exposing cells to an ammonium chloride (i.e., weak acid) solution, neutral ammonia in the extracellular space diffuses into the cytosol across the cell membrane in a concentration-gradient manner, while impermeable ammonium ions remain in the cell exterior. Consequently, a proton is generated in the extracellular microenvironment for rebalancing the NH_3_/NH_4_^+^ equilibrium. This is recorded with the ISFET as a negative overshoot in pH. When the cell microenvironment reaches a steady-state condition, the extracellular pH transient disappears. A positive pH transient occurs after flushing the ammonium chloride solution from the culture media because cell-charged ammonia is diffused out by the concentration-gradient, followed by temporarily rebalancing the NH_3_/NH_4_^+^ equilibrium by consuming protons in the extracellular space. The differential of the potentiometric pH signal is distinct (ΔpH~1 at 10 mM NH_4_Cl) and reproducible (RSD < 5%) during the intervals of weak acid/base loading and unloading. Repeated exposure of ammonium chloride or sodium acetate solutions (~10 mM) cause no apparent acute cytotoxicity during the assay for up to several hours [57]. A variety of cell types can be applied to the pH perturbation assay. Measurements with floating cells, such as T lymphocytes, are possible by functionalizing the ISFET surface with a biomembrane-anchoring molecule [58]. The signal time course is slightly influenced by the cell type, cell adhesion area, cell density, and formation of cell–cell contacts. On the other hand, the pH perturbation assay has some drawbacks toward a wide range of applications. First, long-term measurements with superfusion have increased risks for cell detachment. Second, it is not clear that the pH perturbation can be obtained using thick samples of cell multilayers and ex vivo tissues. Third, numerical analysis is required for quantitatively understanding the perturbation signal.

## 4. Detection of Pore Formation on Biomembranes

**Figure 2:**
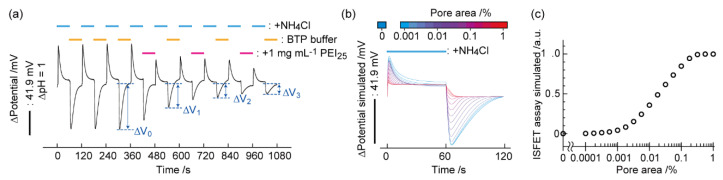
Biomembrane injuries decreased the pH overshoots. (**a**) Time course of the ISFET potential during the intervals of NH_4_Cl loading and unloading with three 1-min exposures of 1 mg/mL [PEI] to the cells. (**b**) Simulation of the ISFET signal during the NH_4_Cl treatment at various ion-accessible pore area percentages (0–1%) of the plasma membranes. (**c**) The reduction rate of the pH overshoot (1−Δ*V*/Δ*V*_0_) as a function of the pore area on the plasma membranes. Reproduced from [46] with permission by Elsevier.

Considering the pH overshoots occur by the semi-permeability of healthy biomembranes at the point of loading and unloading of weak acid/base in the cell microenvironment, a new method for detecting leaky biological membranes was developed (Figure 2) [46]. Namely, ISFET-based active pH sensing was applied for the evaluation of cell membrane damages induced by surfactants or a nanocarrier. A model study using HepG2 cell cultures on an ISFET demonstrated an irreversible decrease in the pH overshoot at the point of ammonia exchange following exposure of the cells to poly(ethyleneimine) (PEI), which a common gene transfer reagent. Cationic PEI forms a pore in anionic biomembranes by pulling the polar headgroup toward the hydrophobic core in the bilayer [59,60]. Therefore, the reduced pH overshoot can be interpreted as elimination of the imbalanced NH_3_/NH_4_^+^ equilibrium because of the free permeation of NH_4_^+^ and H^+^ through the pores on PEI-treated biomembranes. The normalized ISFET signal was determined by the degree of pH perturbation before (Δ*V*_0_) and after one-, two-, and three-time exposures (Δ*V_i_*) of a reagent as: (ISFET signal) = (Δ*V*_0_ − Δ*V_i_*)/Δ*V*_0_. The interpretation is supported by a simulation, in which the pH overshoot disappears when the total pore area exceeds 0.1% the whole cell surface.

**Figure 3:**
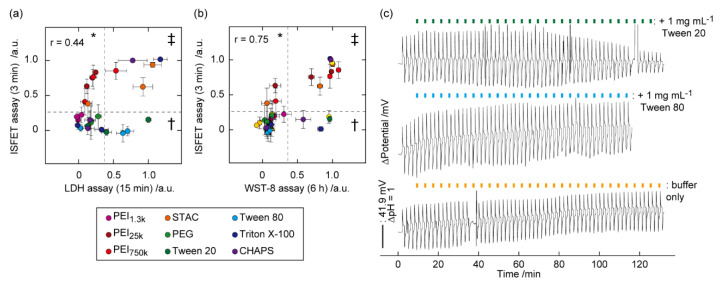
Cluster analysis using the ISFET vs. conventional assays and cell apoptosis detection. (**a**) Scatter plots between the ISFET (3 min) and LDH assays (15 min). *, †, and ‡ represent the ISFET^+^/LDH^−^, ISFET^−^/LDH^+^, and ISFET^+^/LDH^+^ regimes, respectively. Data points identify the two signals at set concentrations with mean ± SD (*n* = 3). LDH signals were normalized by those obtained at 1 mg/mL Tween 20 for 15 min. Colored symbols show chemical species. Dashed lines represent the thresholds. Correlation coefficient: *r*. (**b**) A scatter plot between the ISFET (3 min) and WST-8 (6 h) assays. *, †, and ‡ represent the ISFET^+^/WST-8^−^, ISFET^−^/WST-8^+^, and ISFET^+^/WST-8^+^ regimes, respectively. (**c**) Time course of the ISFET signal during intervals of NH_4_Cl exchanges with/without multiple exposures of the cells to 1 mg/mL Tween 20 and Tween 80. Reproduced with modification from [57] with permission by the Royal Society of Chemistry.

The ammonia-induced active pH sensing for a biomembrane toxicity assay has sensitivity and specificity in the detection of molecularly sized transmembrane pores on the cell membranes because small proton and ammonium ions with a Stokes radii (*R_H_*) < 0.33 nm are an indicator of the leakiness of the biomembrane. This is in sharp contrast with the conventional indicators for membrane toxicity assays, such as calcein dye of *R_H_*~0.74 nm, hemoglobin of *R_H_* > 3.1 nm, and lactate dehydrogenase (LDH) of *R_H_* > 4.2 nm [61,62]. In fact, the ISFET-based assay detects subtle damage of biomembranes that was not detected by LDH leakage assays for membrane toxicity (Figure 3). The results spur us to determine the biomembrane leakages at molecular levels caused by interactions with the nanocarrier and nanomaterial. Information about cell–nanocarrier interactions with molecular definiteness will aid the development of efficient and safe nanocarriers. 

The signal for ammonia-induced pH perturbation is robust against buffering agents in the cell microenvironment [46]. The pH overshoots (ΔpH~1) are not affected by the buffering effect of chemical reagents surrounding cells or proton transporter activities on the cell membranes. Therefore, the ISFET assay has wide applicability to various reagents and cell types. Moreover, the features on high sensor resolution (~40 μV for ΔpH~1.0 × 10^−3^), downsizing, and integration for metal oxide semiconductor-based transistors with the aid of microfabrication technologies could introduce multi-parallel cytotoxicity testing with single-cell resolution. Potentiometric pH measurements using available commercial ISFETs with a superfusion system require only a small number of cells (~10 whole cells), because of the small sensing area (10 μm × 340 μm). Alternatively, existing cytotoxicity assays require thousands of cells per well of a microtiter plate.

## 5. Identification of Biomembrane Injury Type and Cell Death

Biomembrane toxicity assays are frequently used for characterizing the safety of engineered molecules and materials. Cell membrane injuries are typically pore formation, polarity alteration, and disruption (i.e., membrane lysis) [44,63,64,65]. Identification of biomembrane damage is crucial for understanding cell–nanomaterial interactions. However, no existing method could classify biomembrane injuries. Conventional biomembrane toxicity assays, including the calcein/LDH release assays and hemolysis assay, only measure the cumulative amount of indicators or biomarkers released from the cytosol across the leaky plasma membrane of dying or dead cells for an extended period.

As mentioned above, the degree of pH perturbations specifically responds to the pore-forming activity by exogenous reagents because hydrated ions (NH_4_^+^ and H^+^) only pass through transmembrane pores [57]. On the other hand, amphiphilic protein indicators (LDH and hemoglobin) also bypass damaged biomembranes by fusion mechanisms and leakage through large pores. Therefore, the combination of the ISFET assay with conventional membrane toxicity assays was able to classify the type of biomembrane injuries. For example, the scatter plots from the ISFET and LDH assays were categorized into four regimes by setting thresholds (Figure 3a). The double-negative and double-positive regimes indicate intact biomembranes and membrane disruption or lysis, respectively. The ISFET^+^/LDH^−^ regime, which was assigned to cationic reagents, indicates membrane permeability to small NH_4_^+^ and H^+^ (*R_H_* < 0.33 nm), and membrane impermeability to large LDH (*R_H_* > 4.2 nm). This is because cationic reagents form pores sizes smaller than LDH on anionic biomembranes via electrostatic interaction [59,60]. While, the ISFET^−^/LDH^+^ regime, which was assigned to non-ionic or anionic surfactants, was interpreted as membrane impermeability to hydrated NH_4_^+^ and H^+^, and membrane permeability to amphiphilic LDH. The phenomenon is understood as LDH leakage by the fusion mechanism without forming transmembrane pores. The fusion is driven by polarity changes of the biomembranes because of the partitioning of non-ionic surfactants into the lipid bilayers. A similar explanation can be used for the combined analysis of results from the ISFET and calcein assays. Notably, the simple combination of existing assays (without ISFET) was unable to classify the biomembrane injuries.

## 6. Identification of Type of Cell Death

In addition to classifying cell membrane injuries, it is important to understand how cells die as a result of external stimuli, which is key to designing and fabricated safety nanocarriers. Acute cell death mainly occurs by necrosis or apoptosis at different time scales. A severe biomembrane injury leads to detrimental necrosis followed by proinflammatory responses. Apoptosis is categorized in programmed cell death and leads to anti-inflammatory responses. However, conventional cytotoxicity assays only report the results following cell exposures to exogenous reagents and stimuli at a certain endpoint. To address this issue, scatter plots combining the results of the ISFET and commercial cytotoxicity (WST-8) assay were used (Figure 3b) [57]. A detailed analysis using the correlation diagram revealed the cytotoxicity mechanisms. The ISFET^+^/WST-8^+^ cluster was assigned to necrotic cell death induced by irreversible membrane leaking [66]. The ISFET^−^/WST-8^+^ cluster indicates the cytotoxicity induced by damages to subcellular compartments without inducing the biomembrane leakage. The ISFET^+^/WST-8^−^ cluster indicates minor biomembrane damages that can be recovered without causing cytotoxicity.

The process toward cell apoptosis was detected by ISFET monitoring of the pH perturbation for an extended period (~2 h). Apoptosis is the time-dependent programmed cell death mediated by caspase enzymes in the cytosol, leading to gradual disordering of biomembranes (eat-me signaling), followed by eventual clearance by phagocytes. This is in sharp contrast with instant membrane injury during necrosis [67]. The ISFET signal started to respond after 1 h of exposure of cells to an apoptotic inducer (Tween 20), corresponding to increased activity of caspase-3 as an apoptosis marker (Figure 3c) [57]. In contrast, cell exposures to Tween 80, which is a molecular analog to Tween 20, but not an apoptotic inducer, did not cause a time-dependent ISFET signal. Therefore, the ISFET assay for an extended period is effective for distinguishing apoptosis-mediated biomembrane disruptions from direct biomembrane injuries.

**Figure 4:**
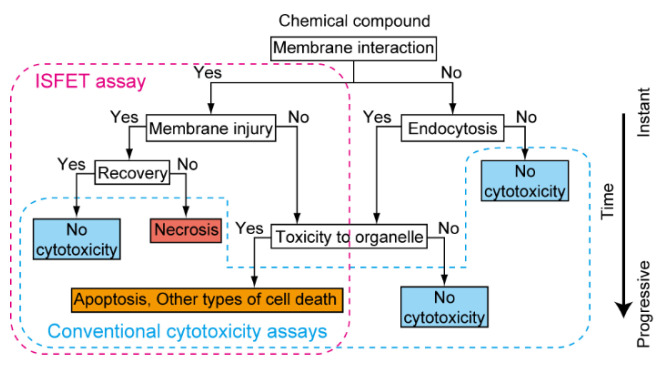
Flow chart showing an invasion of a toxic chemical compound into various cytosolic compartments and the assays that monitor different aspects of time-dependent cytotoxic processes. An invasion of toxicant to the plasma membranes or organelle leads to eventual cell death via different pathways. The ISFET assay detects plasma membrane leakage instantaneously or apoptosis-mediated membrane disorder in several hours. Conventional assays monitor the consequence of toxic activity in minutes to days. Reproduced from [57] with permission by the Royal Society of Chemistry.

Therefore, the ISFET assay complements biomembrane and cytotoxicity assays because of the sensitive and specific detection of small transmembrane pores on the cell surface in real time. The unique features enable us to identify the type of biomembrane injury and the cause of cell death when the analysis is combined with conventional techniques (Figure 4).

## 7. Understanding the Permeation Mechanism of Nanocarrier

Nanomaterials are promising for their use as nanocontainers for DDS. In recent years, some nanomaterials were identified to enter the cell cytosol without energy-dependent cellular uptake mechanisms [68,69]. Oligopeptides, such as the transactivating transcriptional activator (TAT: GRKKRRQRRRPQ) and octa-arginine (R8: RRRRRRRR), are known as cell-penetrating peptides (CPPs) because of their high permeability to live cell cytosols [70]. A water-soluble amphiphilic random copolymer comprising 30 mol% 2-methacryloyloxyethyl phosphorylcholine (MPC) and 70 mol% *n*-butyl methacrylate (BMA), poly(MPC_30_-*r*-BMA_70_) (PMB30W) was found to penetrate the cell membranes in an energy-independent manner without acute cytotoxicity [71,72]. MPC is a methacrylate monomer bearing a zwitterionic phosphorylcholine group in the side chain. PMB30W is a phospholipid-mimicking polymer because of its similar chemical structure to phosphatidylcholine as a main constituent of the lipid bilayers [73]. The energy-independent translocation may improve the efficacy of DDS by bypassing endosomal entrapments during the endocytic processes [74].

Direct penetration of nanomaterials may occur by the creation of transient pores on the cell surface or via fusion with the lipid bilayers [75]. Alternatively, the direct interaction with cell membranes may cause cytotoxic or biocidal effects by breaching the barrier functions. Therefore, an in depth understanding of the underlying mechanisms of non-endocytic internalization of these nanomaterials is important for developing safe and efficient nanocarriers. However, the mechanisms remain elusive because of the lack of sensing methods for detecting the molecularly sized nanopores on the biomembranes. As a result, the ISFET-based pH perturbation assay was used for the analysis of cell-nanomaterial interaction. The ability to sense the formation of pores as small as a proton (*R_H_* < 0.33 nm) can provide solid experimental evidence for the transport mechanism of nanomaterials [46]. Additionally, the combination of the ISFET assay with conventional membrane toxicity and cytotoxicity assays help identify the type of biomembrane injury and cell death as mentioned above [57].

**Figure 5:**
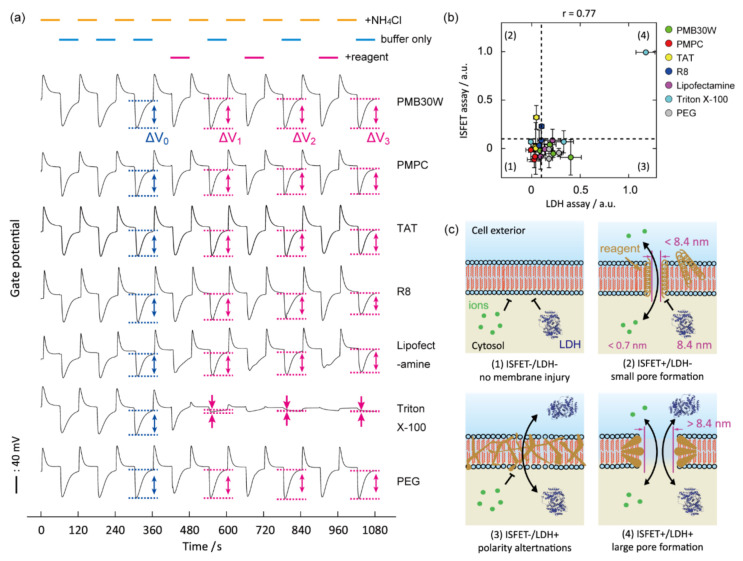
Cluster analysis between the ISFET and LDH assays for identifying the energy-independent permeation mechanism of PMB30W. (**a**) The pH perturbations during repeated exposures of a panel of reagents to cells using a superfusion system. (**b**) Scatter plot between the ISFET and LDH assays. Data points identify the two signals for the samples at set concentrations with mean ± SD (*n* = 5). Dashed lines represent the thresholds defined at 0.1. (**c**) Schematic illustrations showing the types of biomembrane injuries based on the assignment of the four regimes in the correlation diagram. Reproduced with modification from [76] with permission by the American Chemical Society.

Energy-independent internalization of PMB30W was analyzed using an ISFET-based pH perturbation assay [76]. The ISFET signal was stationary following exposures of membrane-permeable PMB30W, a transfection reagent Lipofectamine^®^, membrane-impermeable PMPC, and poly(ethylene glycol) (PEG) (Figure 5). The results indicate PMB30W did not form pores. Alternatively, the ISFET signal responded to TAT and R8 exposures, which indicates proton leaks through small pores. Notably, the pore formations by TAT and R8 were not detected by conventional techniques such as the LDH assay and electrochemical impedance spectroscopy. The scattered plots between the ISFET and LDH assays were classified into the four previously described regions. The ISFET^+^/LDH^−^ (2) regimes for TAT and R8 indicate the formation of molecularly sized pores, which are permeable for H^+^ (*R_H_* < 0.33 nm) and impermeable to LDH (*R_H_* > 4.2 nm). This is the same response as the PEI exposure. In contrast, ISFET^−^/LDH^+^ (3) for PMB30W, Lipofectamine, and PEG was interpreted as chemically induced structural disorders or polarity alterations of biomembranes. PMB30W has an *n*-butyl group, which is less hydrophobic than the fatty acid groups in phospholipids. Therefore, the polarity of the cell membrane could be altered when PMB30W hydrophobically interacts with the lipid bilayer cores. Permeability is expressed as the product of the diffusion coefficient and solubility coefficient. The solubility coefficient of the altered cell membranes differs depending on the solute. Hydrated ions are more hydrophilic than proteins, so they did not dissolve in the altered biomembranes. Therefore, the polarity change allows for permeation of LDH as an amphiphilic enzyme while maintaining the ion-barrier functions of biomembranes. This interpretation aligns with the simulation results [77]. In conclusion, PMB30W entered cells by the amphiphilicity-induced membrane fusion mechanism, not by pore formation.

The same analytical technique was used for other nanocarriers of sulfobetaine polymers that can directly penetrate into cells. Zwitterionic sulfobetaine polymers have bio-inertness and stimuli-responsiveness against temperature, pH, and salts, leading to their application for DDS. Four sulfobetaine polymers with different main chains and cationic moieties were compared [78]. The cluster analysis results indicate that the permeation mechanisms depend on small differences in the chemical structure of the four polymers. Specifically, the intramolecular and intermolecular interactions, such as dipole–dipole, hydrophobic, π–π, NH−π, and cation−π interactions, between the polymer chains are the main drivers for non-endocytic internalization with different mechanisms. 

The method combining the ISFET and LDH assays was further used to explore the effects of lipid-based and polymer-based transfection reagents on the permeability of model endosomal membranes [79]. Commercial Lipofectin™ and in vivo JetPEI^®^ transfection reagents exhibited pH-dependent pore-forming activity under physiological and endosomal pH conditions. Lipid-based Lipofectin™ created proton-permeable small pores. In contrast, polymer-based in vivo JetPEI^®^ caused LDH-permeable large pores. These results are consistent with previous findings that polymer-based cationic nanocarriers achieve endosomal escape through pore formation rather than the proton sponge effect [80,81]. In summary, the ISFET-based pH perturbation assay is expected to help reveal translocation mechanisms of a wide range of nanocarriers.

## 8. Detection of the Breach of the Tight Junction on the Epithelial Cell Layer

In addition to cell membranes, TJs are another form of biological barriers found in intercellular gaps of endothelial and epithelial cell layers, including the BBB. TJs, in addition to other form of cell–cell adhesions, are essential in multicellular organisms for partitioning the interior and external world. The TJ barriers enable nutritional reabsorption in the small intestine and the formation of ion gradients in sensory organs. Therefore, breaching epithelial barriers causes various diseases and infection. TJ cancellations are also involved in cancer metastasis mediated by EMT. In DDS, delivering the nanocarrier-payload complexes through epithelial barriers is a challenge. Temporal breaches of TJ barriers are efficient for translocation; however, they create cytotoxicity issues. The drug delivery via an energy-dependent transcytosis mechanism requires no TJ breakdown; however, it has limitations in the design and application of nanocarrier-payload combination. Therefore, the development of a new transport system that can bypass the epithelial barriers is essential for safe and efficient DDS.

To develop a new transport method in DDS, the evaluation of a nanocarrier–epithelial barrier interaction is necessary. Conventional methods for evaluating epithelial barriers in vitro are trans-epithelial electrical resistance (TEER) and permeability tests. TEER provides information about cell adhesion, migration, and proliferation by measuring AC impedance [82,83]. The resistance and capacitance components are determined from ionic currents through the epithelial monolayers. TJs can be interpreted as resistance by modeling the data with an appropriate equivalent circuit. Alternatively, permeability tests use a low molecular weight, cell-impermeable dye for measuring the permeation through paracellular pathways [84,85]. Although this is simple and common, permeability tests cannot detect minor TJ breakdowns that cause leakage of molecules smaller than the indicator. Moreover, permeability tests suffer from low spatiotemporal resolution because of the monitoring of macroscopic process of mass transport. Therefore, an alternative method for sensing TJ breakdown with high sensitivity is required.

**Figure 6:**
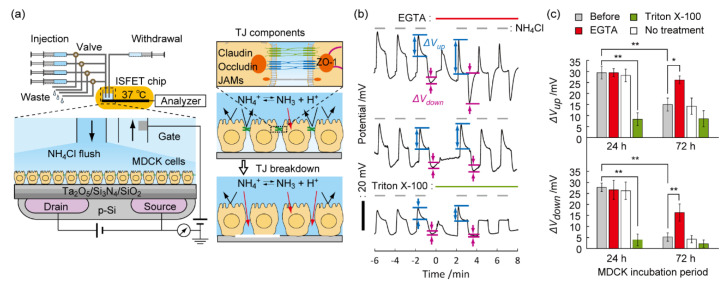
Analysis of barrier functions of epithelial cells with TJs by a pH perturbation assay. (**a**) Schematic diagram showing a system combining ISFET and automated fluidics. Ions permeate cell gaps as a result of TJ breakdown. TJ is mainly composed of claudin, occludin, JAMs, and ZO-1. (**b**) Time course of the gate potential in MDCK cell cultures at 72 h during the NH_4_Cl-induced pH perturbation assay with/without exposure to 1 mM EGTA or 1 mg/mL Triton X-100. (**c**) Δ*V*_up_ and Δ*V*_down_ of MDCK cells cultured for 24 h or 72 h following treatment with EGTA or Triton X-100. Mean ± SD (*n* = 3). * *p* < 0.05, ** *p* < 0.01. Reproduced with modification from [86] with permission by the American Chemical Society.

Prompted by the blocking features of TJs for small ions, the ammonia-induced pH perturbation technique was used for the non-invasive evaluation of TJ barriers in Madin–Darby canine kidney (MDCK) cells. As a proof-of-concept, the ISFET signal was measured following calcium-chelator or cytotoxin exposure to model epithelial monolayers with TJs on the gate insulator [86]. In contrast to decreases in the pH overshoot by biomembrane lysis using a nonionic detergent Triton™ X-100, the degree of pH perturbation was enhanced by specifically breaching TJ barriers using a calcium-chelator ethylene glycol tetraacetic acid (EGTA) (Figure 6). Numerical analysis revealed that the increased permeability of ammonium ions at the paracellular pathway by TJ breaches enhanced the pH overshoot. Therefore, TJ breakdown can be discriminated from biomembrane damage on the epithelial monolayers by monitoring the amplifying or damping trend in pH perturbation. This is a unique feature of the ISFET assay because the conventional TEER and permeability assays cannot differentiate between the two phenomena. Moreover, a small proton (*R_H_* < 0.33 nm) was used to detect the ion barrier breakdown with high sensitivity. The ISFET signal responded to the addition of CPE with a limit of detection (LOD) of 0.03 μg/mL, which was 13-fold less than the LOD of TEER (0.4 μg/mL). Moreover, the effects of the extracellular matrix and a TJ potentiator on the TJ formation process of MDCK cells were successfully evaluated by the ISFET assay [87]. The advanced sensitivity and specificity for examining TJ barriers may create applications including the development of transepithelial nanocarrier, quality control of engineered epithelial tissue, and screening of TJ-targeting drugs.

## 9. Conclusions

The active pH sensing method was developed to overcome the time course of signaling drift in conventional ISFET-based cell assays. The phenomena of pH perturbation induced by flushing weak acid/base in the cell microenvironment was used for the evaluation of biological barriers, such as cell membranes and TJs, on the gate insulator of ISFET. The high sensitivity and specificity to leakages through these barriers originated from the sensing of the smallest proton indicator. Unlike other techniques, the observation of proton leakage provides evidence of the nanopore formation on biomembranes and TJ breaches in the cell gaps, elucidating the permeation mechanisms of drug nanocarriers through the barriers at molecular levels. Moreover, a combination with conventional assays helps identify the type of biomembrane damage and the cause of cell death. As a CMOS-compatible fabrication process, ISFET sensors can be simply integrated into miniaturized high-density sensor arrays in microfluidic chips. Future applications of multi-parallel and single-cell analysis are expected.
